# Supplementary material for: Rapid turnover of life-cycle-related genes in the brown algae
Source: Genome Biol. 2019 Feb 14;20:35. doi: 10.1186/s13059-019-1630-6 (PMC6374913; doi:10.1186/s13059-019-1630-6)
Supplement: Supplementary file 3 — Protein alignments, corresponding Gblocks html file and CDS sequences for the lineage conserved gametophyte-biased genes. (ZIP 2109 kb) [file 13059_2019_1630_MOESM3_ESM.zip › Con_GA_alignments/Ec-17_003220.aa_ali.fasta-gb.htm]

Ec-17\_003220.aa.fasta.trans.aa\_ali.fasta


## Gblocks 0.91b Results

Processed file: **Ec-17\_003220.aa.fasta.trans.aa\_ali.fasta**  
Number of sequences: **2**  
Alignment assumed to be: **Protein**  
New number of positions: **285** (selected positions are underlined in blue)

```
                         10        20        30        40        50        60
                 =========+=========+=========+=========+=========+=========+
Ec-17_003220     MSVPTLGTKALFLPNRKGTLKRDSNRRAKAHRMALQSVRGDVDTREPASMKFSRGQLVDM
MSTRG.37568.1    ------------------------------------------------------------
                                                                             


                         70        80        90       100       110       120
                 =========+=========+=========+=========+=========+=========+
Ec-17_003220     KKLQQEAARAEHVEKCNRKLVDKMMMIMQGLCGTIVSEQTFPDFNPGTLNYRIRQRELVR
MSTRG.37568.1    ------------------------------------------------------------
                                                                             


                        130       140       150       160       170       180
                 =========+=========+=========+=========+=========+=========+
Ec-17_003220     IDRDNLALMHRLETASSGYNKALVKPKYFRQPHMGLFPRSEQSVKSAGSKSRKRIRPGGP
MSTRG.37568.1    ------------------------------------------------------------
                                                                             


                        190       200       210       220       230       240
                 =========+=========+=========+=========+=========+=========+
Ec-17_003220     PTAPSCGSEGRAPSPSFRAGCGGDGGGRSDDGGLGWGASVTSALTEPSSFPTTTLGKFHD
MSTRG.37568.1    ------------------------------------------------------------
                                                                             


                        250       260       270       280       290       300
                 =========+=========+=========+=========+=========+=========+
Ec-17_003220     ASSPASVRKNSGGWRESGADAHASELRIDEPGDLGGGDGGGGVDGGSVHPATGLARTPHP
MSTRG.37568.1    ------------------------------------------------------------
                                                                             


                        310       320       330       340       350       360
                 =========+=========+=========+=========+=========+=========+
Ec-17_003220     PSISSRATCGSLSGKASSKLRKCRRLARPTAAKPGATVFQGGVRIGDDYAIVIVTRATEG
MSTRG.37568.1    --MHSDVPTLSTSGKLPSTLQKCRRLMRVNAAKPGAMVFQGGVRIGDEYAMMTVTRTTEG
                     ########################################################


                        370       380       390       400       410       420
                 =========+=========+=========+=========+=========+=========+
Ec-17_003220     FGDNGTQGFRIKAYFPLSVSVYTLGVSAEHLPVFTAGSHTAVRRLFQTGDKSRGKWEGVV
MSTRG.37568.1    FGEDGTQGFRMRAYCPLSVAVYSLGISASDLPVYTSGSHTAVRRMFQAGEKSRRKWEGVA
                 ############################################################


                        430       440       450       460       470       480
                 =========+=========+=========+=========+=========+=========+
Ec-17_003220     ACLELRPSTDVAGMRKPVESVQSVEGPTPAPSATATGTRSPAVAVPAPAAAGVPSGGMDG
MSTRG.37568.1    GFLELRPSTSDADLRKPVEKVKSSL-ADPTTAGSPPDTVGTGAGSNAPAATAVPLTGLDG
                 #######################     ################################


                        490       500       510       520       530       540
                 =========+=========+=========+=========+=========+=========+
Ec-17_003220     TEERVGAPSTGTAGAATAATVPMAVGVEMVLVPPEDAPVLIVQRLVRGWLSRQQYQSLKR
MSTRG.37568.1    TEETGGE--T-AAALVVPPAVDPPRRLEMVLVPPEDAPVIIIQRLVRGLIARDTYARLKR
                 ######      ################################################


                        550       560       570       580       590       600
                 =========+=========+=========+=========+=========+=========+
Ec-17_003220     SVIVIQAAIRGWKYRVAKRKHEAATKIAAVSRGKRCRNRVKRERAARGIQKRTRSWLRKR
MSTRG.37568.1    SIVVIQAAVRGWKYRMAEWKHSAATKIAAVTRGKRCRTRVKQERAARGIQKRTRLWLRKR
                 ############################################################


                        610       620       630       640       650       660
                 =========+=========+=========+=========+=========+=========+
Ec-17_003220     DVEKDRAAKQIQAAVKRRRKRLGQDMRHVVQKEESSVLSRRGILPCNIINSKDAACRRGR
MSTRG.37568.1    EX----------------------------------------------------------
                                                                             


                        670       680
                 =========+=========+====
Ec-17_003220     CAWPRSRPQATTTVTQIRRDLDRQ
MSTRG.37568.1    ------------------------
```

```
Parameters used
Minimum Number Of Sequences For A Conserved Position: 2
Minimum Number Of Sequences For A Flanking Position: 2
Maximum Number Of Contiguous Nonconserved Positions: 8
Minimum Length Of A Block: 10
Allowed Gap Positions: None
Use Similarity Matrices: Yes
```

```
Flank positions of the 3 selected block(s)
Flanks: [305  443]  [449  486]  [493  600]  

New number of positions in Ec-17_003220.aa.fasta.trans.aa_ali.fasta-gb:  285  (41% of the original 684 positions)
```
